# Canadian demand and access to corneal transplantation: a provincial comparison

**DOI:** 10.1007/s10561-021-09968-y

**Published:** 2021-11-12

**Authors:** Christine Humphreys, Kyle Maru, Sonia N. Yeung, Guillermo Rocha, Clara C. Chan

**Affiliations:** 1 Eye Bank of Canada - Ontario Division, Toronto, ON Canada; 2 Canadian Blood Services, 1800 Alta Vista Drive, Ottawa, ON K1G 4J5 Canada; 3 Department of Ophthalmology, University of British Columbia, Vancouver, BC Canada; 4 Eye Bank of British Columbia, Vancouver, BC Canada; 5 Department of Ophthalmology, University of Manitoba, Winnipeg, MB Canada; 6 Department of Ophthalmology and Vision Sciences, University of Toronto, Toronto, ON Canada

**Keywords:** Corneal transplantation, Access, Demand, Canada, Wait times, Infrastructure

## Abstract

To gather information from stakeholders involved in corneal donation and transplantation to inform discussion at the “National Consensus Forum on Improving Cornea Donation and Transplantation Access in Canada” held in February 2020, survey questions were posed to eye banks, transplanting ophthalmologists and organ donation organizations across Canada to learn more about demand, wait times, and access to tissue for transplant. The survey response rate was one hundred percent (100%) for eye banks and organ donation organizations while 64 percent (64%) of transplant ophthalmologists provided feedback. A number of opportunities for improvement were identified including: demand forecasting; infrastructure and strategies to align supply with demand; data collection and benchmarking of wait times for assessment and transplant to support consistency, equitability and transparency in access; and national collaboration in the development of a data strategy to accurately measure demand and access to cornea transplants in a consistent manner across all provinces to facilitate equity in access nationally.

## Introduction

**Fig. 1:**
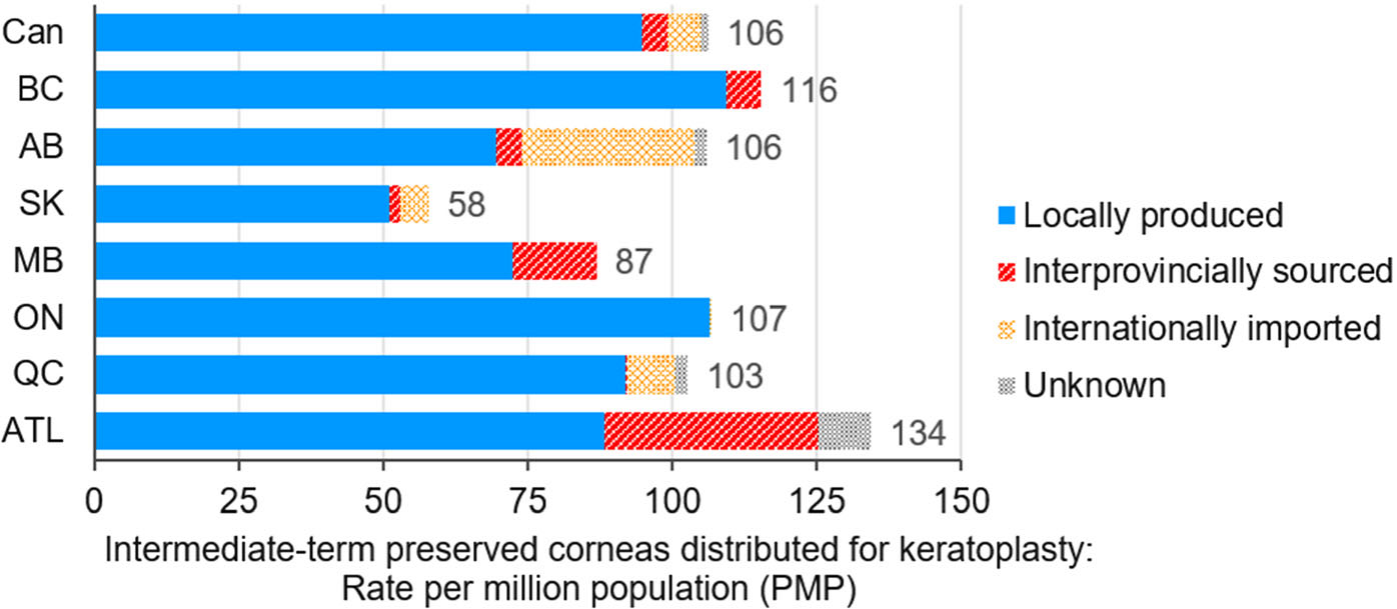
Bar graph showing population-based rates of intermediate-term preserved corneas distribution for keratoplasty by province in 2019. Results show substantial provincial disparities in availability of corneas for transplant and differing needs to source corneas out-of-province. To meet demand the majority of eye banks are required to supplement in-province cornea graft production to some degree with corneas sourced interprovincially or internationally.^1^

The need for corneal transplantation in Canada is understood to be increasing each year (Canadian Blood Services 2020a). Contributing factors may include an overall aging population, changes in surgical techniques which allow for better outcomes, wider indications for surgery, and a reduction in the length of time for surgical procedures with the implementation of pre-cut and pre-stripped tissue; however, the growth in cornea transplantation in Canada over the last 6 years has been stagnant, with data collected by Canadian Blood Services’ Eye and Tissue Data Committee indicating that there were 3901 corneal transplants in 2019 compared to 3891 corneal transplants in 2014 (Canadian Blood Services in press). Most eye banks from across Canada are required to bring in corneas from out of province to meet surgical demand for corneal transplantation (Fig. 1), some more than others (Canadian Blood Services 2020b).

Canadian Blood Services and the Canadian Ophthalmological Society partnered to engage the cornea donation and transplantation community through the *National Consensus Forum on Improving Cornea Donation and Transplantation Access in Canada* held on February 9–10, 2020 in Toronto (see Bae et al. 2021). This forum was purposed at determining gaps in the Canadian system; identifying barriers and facilitators for change; and developing recommendations to improve access to, and equity in, cornea transplantation in Canada. Significant effort was made to gather evidence that would prepare and inform meeting participants for discussion and debate. This included the results of a survey of Canadian eye banks, organ donation organizations and corneal transplant surgeons.

### Objective

The goal of this work is to better understand the current situation in Canada with respect to access to cornea transplantation in terms of the demand, with particular reference to our visibility into patients waiting and the general landscape of demand for cornea transplantation in Canada. More specifically, the primary objective is to provide a descriptive analysis of data collected from organ donation organizations, eye banks, and transplanting ophthalmologists relating to their understanding of the need for cornea transplantation in Canada and the degree to which the current donation and transplantation system is able to meet that need. Secondary objectives stemming from this analysis are to provide evidence in support of meaningful gaps in access to transplantation, stakeholder perspectives, and standardization of practice in demand management.

### Scope

Included within the work are the perspectives of representatives from all eye banks and all organ donation organizations in Canada, as well as all transplanting ophthalmologists in Canada, at the end of 2019, in addition to quantitative data on national system performance provided by all Canadian eye banks as they pertain to access to, and demand for, cornea transplantation.

With the specific focus of this work being on access and demand, perspectives on related themes such as cornea supply, donor/cornea utilization, program operations, and other metrics related to system improvement are outside the scope of the present analysis. Although data was collected on these themes as part of the broader information gathering project that was conducted in preparation for the 2020 Cornea Consensus Forum, results specifically relating to these themes are not analyzed in this work. Similarly, while international representatives participated in the 2020 Cornea Consensus Conference, the scope of this analysis is limited to Canadian results. It is nonetheless hoped that the results may provide insights that are of value in other national contexts and the general eye banking community as a whole.

Perspectives of other stakeholder groups, including patients, donor families, program administrators, coordinators, and others involved in the cornea donation and transplantation process are not encompassed in this analysis; however, representation from these groups was incorporated at the 2020 Consensus Forum Conference that was informed by the results of this research. Similarly, perspectives on related themes such as increasing cornea supply, donor and graft utilization, and other related components of the national donation and transplantation system are not included within the scope of this analysis but were included as discussion themes at the 2020 Consensus Forum Conference.

### Material and methods

To gather information from stakeholders involved in corneal donation and transplantation to inform discussion at the Consensus Forum Conference in 2020, survey questions were posed to eye banks, transplanting ophthalmologists and organ donation organizations across Canada to learn more about demand, wait times, and access to tissue for transplant. Data was collected through three separate surveys tailored to each respondent group and distributed in November and December of 2019 through the Interceptum online survey platform. Respondents also had the option of providing their responses by e-mail using an equivalent template if requested, although none of the respondents elected to submit result in this manner.

Each survey was created to collect targeted stakeholder information on demand and access as it relates to cornea transplantation, as well as other related topics. Surveys included necessary explanations for informed participation and were pre-tested internally and externally to ensure the questions were unambiguous and easy to understand with sufficient detail to obtain the necessary information, and that questions were not open to interpretation. Ethics approval was not applicable. A survey guide distributed along with the survey explained that the results would be used for discussion at the Consensus Forum Conference and confidentiality of respondents would be maintained. Informed consent was obtained from all individual participants. Furthermore, at the Consensus Forum Conference, attendees signed a consent form that authorized the recording of discussions and collection of data that would later be formatted for publication.

The primary data source for the quantitative and qualitative data in this study was collected using three targeted surveys, each purposed at collecting information from one of three respondent groups: surveys were sent to all Canadian eye banks, organ donation organizations and transplant ophthalmologists. A summary of the question categories and types of data collected is provided in Table 1.

**Table 1:** Summary of survey questions for access and demand themes by data type and by respondent group (denoted by “X”)

Question	Data type	Respondent type		
		Eye bank	ODO	TxO
*Demographics*				
Transplants and types performed	Ordinal & Ratio			X
Province/Organization	Categorical	X	X	X
Organization’s role in system	Categorical	X	X	
*Access Theme*				
Access to tissue for transplant	Ordinal (Likert)	X		X
Allocation process	Categorical	X		X
Fairness in allocation	Categorical	X		X
Factors in allocation	Categorical			X
Waitlist management practices	Categorical			X
Wait time definitions	Categorical	X		X
Estimated wait time	Ordinal	X	X	X
Appropriate wait time	Ordinal (Likert)	X	X	X
*Demand Theme*				
National	Categorical	X		X
Forecasting activity	Categorical & Qualitative	X	X	X
Impact of emerging technologies	Ordinal (Likert)	X	X	X
Clinical Outcomes	Categorical	X		
Support for national registry	Categorical	X		
Role of Prevention	Qualitative			X

[ODO: Organ Donation Organizations, TxO: Transplanting Ophthalmologists]

Participants were also invited to provide qualitative comments in relation to each question/section and for the survey as a whole. In addition to the question sections presented here, participants also provided responses relating to tissue supply and utilization, operational models, and metrics for system performance improvement

Survey data, including both qualitative and quantitative responses from all three groups, was analyzed and presented in summary form to the participants of the 2020 Consensus Forum Conference to inform their discussion.

As an adjunct to the survey data, national annual data from the Eye and Tissue Data Committee was utilized. The Eye and Tissue Data Committee is a committee under Canadian Blood Services, which collects data from all eye banks in Canada that is utilized in annual national reporting. This data is provided to the Committee by representatives of each Canadian eye bank, and undergoes a multi-level validation process with review by eye bank and Canadian Blood Services representatives through the Committee, as well as presentation of provincial and national results by the Canadian Ophthalmological Society for review and discussion, prior to general publication.

### Results

#### Response rate

There was a 100% compliance rate in completed surveys for eye banks and organ procurement organizations.

Nine out of nine eye banks participated including: the Eye Bank of British Columbia, Comprehensive Tissue Centre (northern Alberta), Lions Eye Bank of Calgary (southern Alberta), Saskatchewan Transplant Program, Misericordia Eye Bank (Manitoba), Eye Bank of Canada (Ontario Division), Banque d’yeux du Québec and Banque d’yeux du Centre Universitaire d’Ophtalmologie (Québec), New Brunswick Organ and Tissue Program—Ocular Division, and Regional Tissue Bank (Nova Scotia).

Ten out of ten Organ Procurement Organizations participated including: BC Transplant, Human Organ Procurement and Exchange Program (northern Alberta), Southern Alberta Organ and Tissue Donation Program, Saskatchewan Transplant Program, Transplant Manitoba Gift of Life Program, Trillium Gift of Life Network (Ontario), Transplant Quebec/Hema-Quebec, New Brunswick Organ and Tissue Program, Legacy of Life Organ and Tissue Donation Program (Halifax), Organ Procurement and Exchange Program (Newfoundland).

Nationally, there were 42 survey respondents (64%) from active transplanting ophthalmologists (Table 2).

**Table 2:** Counts of transplanting ophthalmologists surveyed (total invited and number responding) by province

Province	Number of respondents	Number of transplanting ophthalmologists surveyed
Alberta	0	7
British Columbia	6	12
Manitoba	2	2
Nova Scotia/New Brunswick	3	3
Ontario	18	26
Quebec	13	15
Saskatchewan	0	1
*TOTAL*	*42*	*66*

In total, 42 transplanting ophthalmologists from across Canada provided responses to the survey

#### Demand

Demand for cornea transplantation in Canada is typically defined as the number of people approved for and actively awaiting corneal transplant surgery. In the 2019 nationwide survey, 59% of transplant ophthalmologists and 75% of eye banks indicated that in addition to the current waiting lists (demand), there are patients not being referred for cornea transplantation due to their age, or the perception that there are not enough donors and that the wait lists are too long.

Every eye bank, 60% of organ donation organizations, and 51% of transplant ophthalmologists reported challenges in meeting demand. Of interest, the opinions expressed by ophthalmologists did appear to be associated with the size of their respective provincial population, with ophthalmologists from provinces with larger populations being more likely to report that they had ready access to tissue at all times and those from smaller provinces being more likely to report that transplants are booked based upon tissue availability as opposed to patient need or operating room availability.^2^ Notwithstanding this observed pattern, further research would be required to confirm the legitimacy of this finding.

Several systemic gaps were identified that impact most, but not all, provinces: gaps include the lack of visibility into the true level of demand in Canada, lack of comprehensive data or established and standardized processes to collect data around the number of patients waiting for cornea transplant or their waiting time, no formal reporting of demand or waiting times, and no requirement for eye banks to set targets for supply, recovery and processing that align with demand.

Available quantitative data on corneas distributed for transplant suggests very little change over time at a national level, including in 2020. Despite donation and transplantation activity in Canada being curtailed in response to the COVID-19 pandemic in 2020, national-level data suggests that 2020 cornea transplantation activity was approximately equivalent to activity levels from 5 years prior (Canadian Blood Services, in press). When looking at this trend in isolation, it could be argued that the observed pattern of stability reflected a consistent level of demand for cornea transplants; however, the data collected as part of this study would not be consistent with this interpretation.

Instead, the alternative interpretation that would be consistent with our findings is that, because of the aforementioned gaps in meeting demand identified by transplanting ophthalmologists and organ donation organizations, the pattern observed reflects a ceiling effect caused by limitations in supply. That is, while the portion of total demand observable through cornea distribution patterns provides visibility at a baseline level which is limited by graft availability, true demand exceeds supply on a national scale to a degree which is not quantifiable at present. Unfortunately, our visibility into true need, including the ‘dark figure’ of unmet demand, is limited. As such, there is no robust way to accurately determine demand nationally in Canada, in large part because many of the surveyed eye banks report that current waiting lists may not be accurate, while others report having no visibility to current waiting lists, as these are managed by individual transplant surgeons’ offices. Moreover, the results of this study suggest that the degree to which the there exists a supply-demand gap in Canada is not uniform between regions, with some banks potentially having access to sufficient donors to meet or potentially exceed the needs within their jurisdiction.

#### Wait times and wait lists

Corneal transplant surgeons’ offices maintain and/or monitor waitlists in most provinces apart from Alberta and Quebec. Alberta Health Services monitors wait-times for corneal transplant, whereas in Quebec the hospitals perform this function. In the eye banks in British Columbia and Manitoba, the eye bank has access to waitlists. In Saskatchewan, the transplant surgeons add and remove patients from the wait list and the eye bank reports to the Ministry of Health the number of patients waiting, number of patients transplanted and time frames. In Manitoba, the wait lists are monitored and prioritized by the surgeon’s office, however the eye bank and hospital operating room may access wait times through a scheduling system. In Ontario, transplant surgeons maintain their own waitlists and are required to report wait times to Ontario Health (Health Quality Ontario nd). Wait times are publicly reported and available online at the Health Quality Ontario web site for anyone to access. The data comes from the Wait Time Information System™ (WTIS™).

Eighty-five percent of transplanting ophthalmologists reported that patients are seen within 6 months or less; however, 15% indicate wait times of up to 2 years. Forty percent of transplanting ophthalmologists indicated referral times for transplant assessment require improvement, while 45% of transplant ophthalmologists and 75% of the eye banks indicated that wait times from identifying the need for cornea transplant to actual transplant require improvement. Waiting time from referral to seeing an urgent patient is estimated to vary from less than 1 month to more than 6 months depending on the province. Waiting time for non-urgent patients is estimated to vary from one month to more than 2 years depending on the province.

Most transplanting ophthalmologists indicated that they believe there are enough surgeons in their respective provinces to provide appropriate access to corneal transplantation, however 11% indicated that a lack of surgeons contributed to increased wait times in their province.

Most respondents indicated that waiting times from assessment to transplant of less than 1 month would be appropriate for emergent patients, though ophthalmologists estimated twenty percent of emergent patients were not accommodated in that time. Additionally, most felt that waiting times of less than three months would be appropriate for urgent patients, however ophthalmologists estimated twenty-five percent of urgent patients were not accommodated in that time.

New Brunswick, Saskatchewan and Manitoba eye banks estimate the wait for non-urgent patients as 1 to 2 years or longer. Nova Scotia and Alberta eye banks were unable to estimate wait time in their provinces indicating they did not have access to this information as wait lists are managed by the ophthalmologist’s office. Ninety-three percent of ophthalmologists suggested wait times of less than 6 months would be appropriate for non-urgent patients. Forty-five percent of ophthalmologists estimate non-urgent patients are not transplanted within the time frame of 6 months, while 33% of the ophthalmologists indicated non-urgent patients wait between 1 and 2 years for transplant.

Two out of nine eye banks indicated that the criteria defining the urgency of the need for corneal transplant was determined by the transplanting ophthalmologist. The eye banks were not necessarily made aware of what criteria the transplanting ophthalmologists applied to determine whether a case is urgent versus emergent. There are no standardized national definitions, however Ontario has identified targets where emergent surgery should take place within 24 h, and urgent surgery within 30 days of making the decision to transplant.

The surveys identified systemic gaps across most provinces. With one exception (Ontario tracks wait time data), there is no comprehensive data set nor an established and standardized process to collect data on patient access to cornea transplantation. This includes a lack of formal reporting requirements or benchmarks for access in most provinces. Without data on reporting and benchmarks there is a lack of quality controls or accountability processes in place to protect patients and ensure their access is appropriate. Approximately 20% of transplanting ophthalmologists and 50% of eye banks rate access to cornea tissue as poor. The physician and eye bank communities felt overall there was a lack of standardized protocols on the allocation of donated corneas to waiting patients and that currently there are no guidelines to advise this decision; the allocation choice would not only vary between provinces, but within provinces based on surgical practices.

All eye banks reported having an established process to allocate corneas, however 22% of eye banks do not believe the cornea allocation process is as fair and equitable as it could be. Just under 25% of transplanting ophthalmologists indicated that they believed that patient and wait time were a factor in allocation decisions.

#### Prevention

Of the transplanting ophthalmologists who participated in the survey, 51% shared their thoughts on the role prevention should, or could, play in reducing Canadian demand (the remaining participants either indicated that they didn’t know or declined to answer). The survey showed that prevention in the form of enhanced education for contact lens wear, crosslinking coverage, trauma prevention, and early screening/referral could play a role in reducing the demand for transplantation. While 25% of those who responded to this item indicated that they felt prevention could or should play a minimal role and/or noted that dystrophies are not preventable, most indicated support for increased public awareness to prevent activities that can lead to ocular damage such as eye rubbing which worsens the progression of ectatic conditions and poor contact lens hygiene which increases the risk for corneal infections.

### Discussion

#### Methodological limitations

The major strength of our survey study was comprehensive data from 100% of all eye banks and organ donation organizations, and a high return rate of 64% from transplanting surgeons. However, as surveys are more standardized, it can be difficult to drill down to obtain details. Questions tend to be more generalized and can potentially lack depth. We attempted to overcome this by adding the ability for respondents to provide comments and to contextualize their answers. Interpretation of questions can be problematic as well. The other potential weakness of this data set was the likely presence of personal biases held by respondents and the challenge to differentiate between subjective perceptions and objective assessments, particularly in cases where empirical data on which to base their responses was not accessible to respondents.

#### Future directions

Canada has historically faced problems with long wait times for cornea transplant at a national level (Kramer 2013), and following the COVID-19 pandemic, there may be lasting effects on healthcare services around the world as cornea transplant patients face long waiting lists and potential shortages of donors (Ang et al. 2020).

Currently, many provinces have no formal reporting measures for patient demand or waiting times. As well, most eye banks are not required to set targets for supply, recovery, and processing that align with demand. Without these measures for accountability, eye banks facing conditions characterized by product shortages may not be able to meet the needs of patients and surgeons. Survey results indicate that some patients who may benefit from corneal transplant are not listed which suggests that further inquiry into need, as opposed to demand may be necessary.

Tracking surgical demand or need alone does not ensure the system is performing well. Only Ontario and Quebec engage in demand forecasting to align supply with demand. There is an opportunity for national collaboration in the development of a data strategy that will accurately measure demand and access to assessment in a consistent manner across all provinces. Setting benchmarks for waiting times, access, supply, recovery, and processing are an essential component of a data strategy. Developing and advancing a model for forecasting demand would inform operational strategies aligning supply and demand. Additionally, this suggests there may be an opportunity to explore the need for developing national standards for allocation given the variation in wait times by surgeon and province to standardize allocation nationally.

#### Areas of continued research

It should also be noted that, while such measures may serve as an initial step toward better meeting demand in Canada, addressing supply-demand gaps will undoubtedly require that changes be made and new measures be adopted to ensure that the supply of transplantable cornea grafts is adequate to meet demand at a national level. Although an analysis of supply-related factors is outside of the scope of the present examination, one such measure may involve implementing or improving programs and policies for systematic cornea sharing between provinces and jurisdictions to allow improvements in the efficient utilization of extant domestic resources, as well as exploring options for optimizing the utilization and mobilization of these resources; for instance, maximizing recoveries from current potential cornea donor sources. Kramer (2013) suggested that the failure to realize all potential opportunities for cornea donation is a major contributing factor to the difficulties that Canada has historically experienced in meeting demand.

In addition, measures aimed at increasing cornea donor availability may prove fruitful in this regard, such as expansion of efforts to promote cornea donation through public awareness initiatives and related measures. Similarly, legislative changes including the transition to presumed (‘opt-out’) models for consenting donors may influence the availability of cornea grafts for transplant, although given the novelty of this approach in a Canadian context, the net effect of such policies on access to transplantation in Canada has yet to be determined; however, international comparison of donation consent models suggest that corneal procurement rates may be significantly higher in jurisdictions employing an opt-out system as compared with those requiring donors or representatives to give explicit consent (Gain et al. 2016). Although an analysis of measures oriented around maximizing cornea graft supply in Canada and international comparisons are outside of the scope of this analysis, further research exploring these themes is recommended.

In any case, at present feedback from eye banks and ophthalmologists indicate the existence of large discrepancies between provinces with respect to wait times. In most cases, the only data available are estimates provided by surgeons and/or eye banks, which may be inaccurate. To truly understand wait times, and in doing so begin to improve them, a standardized process and comprehensive data set is required to accurately measure those actively awaiting cornea transplantation.

### Conclusions

The survey findings suggest there are several opportunities for improvement, most importantly the enabling of collaboration and sharing of information to improve equity to access nationally. Ideally by optimizing existing provincial infrastructure to develop an interprovincially coordinated and patient-driven supply chain and distribution strategy that aligns supply with demand and ensures patients will be scheduled for transplant based on their need, and not based on when the tissue becomes available. This could also mean developing and advancing a model for forecasting need and demand to inform operational strategies that will ensure that supply aligns with demand.

While there was not support for development of a national waitlist, there is an opportunity for national collaboration in the development of a data strategy that will accurately measure patient need and access to assessment and transplant in a consistent manner across all provinces. Setting benchmarks for waiting times, access, supply, recovery, and processing are an essential component of a data strategy. With a national data strategy there is the potential to address one of the main weaknesses of these surveys: the ability to make data driven decisions as opposed to using qualitative observations.

While each province has waitlists, wait times for both assessment and transplant are not always tracked and criteria for patients being waitlisted and visibility to the waitlists are not consistent nationally. Tracking access to assessment and corneal transplant based on strict and consistently applied criteria is suggested by the survey findings. The provision of data to better understand urgency and demand will allow programs to adjust operations to meet demand and support greater transparency, consistency and equitability in access across Canada, as well as enabling international benchmarking.

Development of a provincial advisory committee for cornea transplantation with links directly to the appropriate government representatives would promote a collaborative focus on nationwide goals, facilitate routine discussions about demand and waitlists, provide a forum for updates on current metrics, and ensure that all provinces are accountable for improving the system to ensure timely patient care.

## Data Availability

All authors had access to the data and approved of the materials. The datasets generated and analyzed during the current study are available from the corresponding author on reasonable request.

## Footnotes

1 *Figure notes: Results are based on data submitted through the Eye and Tissue Data Committee annual national census. Atlantic patients are transplanted in Nova Scotia thus the rate calculation includes populations of all Atlantic provinces (Nova Scotia, New Brunswick, Prince Edward Island, and Newfoundland). Unknown cases reflect cornea distributions for which the procedure type was not available and may include non-keratoplasty procedures. Results presented do not include long-term preserved cornea distribution. Per million population rates based on Statistics Canada population estimates by province as of July 1, 2019 (Table 1710-0086-1). National rate is based on the entire national population, including Newfoundland, Yukon, Northwest Territories, and Nunavut.

2 There is a strong linear association (*r* = 0.93) between mid-year provincial population estimates (Statistics Canada population estimates by province as of July 1, 2019, Table 17-10-0086-01) and mean ratings relating to ophthalmologists’ perceived access to corneal tissue grafts for transplantation (responses coded as 1, 0, and -1 corresponding to the ‘excellent’ ‘adequate’ and ‘poor’ tissue access options).

## References

[CR1] Ang M, Moriyama A, Colby K, Sutton G, Liang L, Sharma N, Hjortda J, Shun Chiu Lam D, Williams GP, Armitage J, Mehta JS (2020). Corneal transplantation in the aftermath of the COVID-19 pandemic: an international perspective. Br J Ophthalmol.

[CR2] Bae SS, Rocha G, Humphreys C, Chan CC, Yeung SN (2021). A national consensus forum on improving cornea donation and transplantation access in Canada. BMC Proc.

[CR3] Canadian Blood Services (2020a) Canadian Eye and Tissue Bank Statistics 2018. https://professionaleducation.blood.ca/en/organs-and-tissues/reports/eye-and-tissue-reports-and-surveys

[CR4] Canadian Blood Services (2020b) Canadian Eye and Tissue Bank Statistics 2019. https://professionaleducation.blood.ca/en/organs-and-tissues/reports/eye-and-tissue-reports-and-surveys

[CR5] Canadian Blood Services (in press) Canadian Eye and Tissue Bank Statistics 2020

[CR6] Gain P, Jullienne R, He Z, Aldossary M, Acquart S, Cognasse F, Thuret G (2016). Global survey of corneal transplantation and eye banking. JAMA Ophthalmol.

[CR7] Kramer L (2013). Corneal Transplant Wait List Varies across Canada. CMAJ.

[CR8] Health Quality Ontario (nd) System Performance: Measuring Wait Times for Eye Surgeries. https://www.hqontario.ca/System-Performance/Measuring-System-Performance/Measuring-Wait-Times-for-Eye-Surgeries. Accessed from 20 January 2021

